# Experimental Evidence Against Taurine Deficiency as a Driver of Aging in Humans

**DOI:** 10.1111/acel.70191

**Published:** 2025-08-11

**Authors:** Vincent Marcangeli, Marina Cefis, Rami Hammad, Jordan Granet, Jean‐Philippe Leduc‐Gaudet, Pierrette Gaudreau, Mylène Aubertin‐Leheudre, Marc Bélanger, Richard Robitaille, José A. Morais, Gilles Gouspillou

**Affiliations:** ^1^ Département Des Sciences de L'activité Physique UQAM Montreal Quebec Canada; ^2^ Département Des Sciences Biologiques UQAM Montreal Quebec Canada; ^3^ Groupe de Recherche en Activité Physique Adaptée Montréal Québec Canada; ^4^ INSERM UMR1093‐Caps Université de Bourgogne, UFR Des Sciences de Santé Dijon France; ^5^ Centre de Recherche de L'institut Universitaire de Gériatrie de Montréal Montreal Quebec Canada; ^6^ Department of Movement Sciences and Training, Faculty of Exercise Sciences University of Jordan Amman Jordan; ^7^ Research Group in Cellular Signaling, Department of Medical Biology Université du Québec À Trois‐Rivières Trois‐Rivières Canada; ^8^ Centre de Recherche du Centre Hospitalier de l’Université de Montréal, Département de médecine Université de Montréal Montreal Quebec Canada; ^9^ Département de Neurosciences Université de Montréal Montreal Quebec Canada; ^10^ Centre interdisciplinaire de recherche sur le cerveau et l’apprentissage (CIRCA) Université de Montréal Montreal Quebec Canada; ^11^ Research Institute of the McGill University Health Centre and McGill University Montreal Quebec Canada; ^12^ Meakins‐Christie Laboratories, Department of Medicine McGill University Montreal Quebec Canada

**Keywords:** aging biomarker, functional capacities, geroscience, mitochondria, mitochondrial function, physical activity, sarcopenia, skeletal muscle, taurine

## Abstract

Taurine deficiency was recently proposed as a driver of aging in various species, including humans. To test this hypothesis, we assessed whether circulating taurine was associated with aging and physical performance in 137 physically inactive and physically active men aged 20–93. No association between circulating taurine levels and age, muscle mass, strength, physical performance, or mitochondrial function was observed, thereby challenging the implication of taurine deficiency as a primary driver of aging in humans.

## Graphical abstract

Circulating taurine concentration is not associated with age, muscle mass, strength, physical performance, or mitochondrial function in humans. Taurine deficiency is therefore unlikely to be a primary driver of aging in humans.

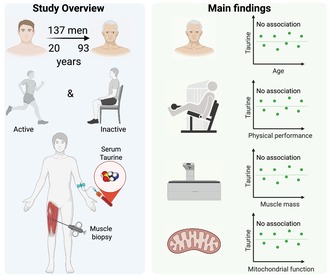

Aging is a highly complex and multifaceted process characterized by a gradual decline in physiological function, physical capability, and resilience, accompanied by an increased vulnerability to disease and mortality (Janssen et al. 2002; Kennedy et al. 2014; Lopez‐Otin et al. 2023; Sen et al. 2016). Despite significant advancements in aging research over the past few decades, the fundamental drivers of aging remain poorly understood. Deciphering the processes that govern aging, with the ultimate aim of extending both healthspan and lifespan, stands as one of the most exciting and critical challenges in modern science.

In an elegant and comprehensive study, Singh et al. recently provided robust evidence positioning taurine (2‐amino ethane‐sulfonic acid; a semi‐essential amino acid) deficiency as a driver of aging in *Caenorhabditis elegans* and mice, and as a determinant of healthspan in both mice and monkeys (Singh et al. 2023). Indeed, circulating taurine levels were found to decline with age in mice and monkeys, and restoration of youthful levels through supplementation was found to increase lifespan in *C. elegans* and mice and increase markers of healthspan in mice and monkeys (Singh et al. 2023). The protective effects of taurine supplementation in mice and monkeys notably included an increase in muscle strength and improvements in body composition, glucose handling, and in multiple hallmarks of aging, including mitochondrial function (Singh et al. 2023). Importantly, the authors also provided data in humans indicative of a decline in circulating taurine levels with aging, albeit the correlation reported seemed mainly driven by individuals aged 20 and under, and participants over the age of 65 were lacking (Singh et al. 2023). Interestingly, low circulating taurine levels were also associated with multiple aging‐related diseases in humans (Singh et al. 2023). Conversely, the authors reported that acute physical exercise increases circulating taurine levels, suggesting that an increase in circulating taurine levels may contribute to the health benefits of exercise and physical activity (Singh et al. 2023). While compelling in worms, mice, and non‐human primates, the study from Singh et al. provided limited investigation of the potential role played by taurine in aging and healthspan in humans, highlighting the need to further test the hypothesis that taurine deficiency is a primary mechanism driving aging in humans. Further highlighting the need for additional investigation in humans, the available evidence indicates that taurine metabolism may differ in mice and humans. In animal models, studies have provided clear evidence that taurine is an important regulator of muscle function (Goodman et al. 2009; Hamilton et al. 2006; Terrill et al. 2016). Taurine supplementation for 2 weeks in rodents has also been shown to increase muscle taurine content by approximately 40% (Goodman et al. 2009). In contrast, a study conducted in humans reported no increase in muscle taurine levels after 7 days of supplementation (4.98 g/day; Galloway et al. 2008). In the setting of exercise performance in humans, the limited available literature is filled with mixed data, with some studies reporting a positive impact of taurine supplementation on physical performance (da Silva et al. 2014; Waldron et al. 2018; Zhang et al. 2004) while others report no improvement (Kurtz et al. 2021; Rutherford et al. 2010; Spriet and Whitfield 2015; Ward et al. 2016). These discrepancies and potential species specificity highlight the need for further research aimed at clarifying whether changes in taurine levels drive aging in humans.

For taurine deficiency to be a driver of aging and healthspan in humans, we reasoned that circulating taurine should progressively decline throughout adulthood and should correlate with indicators of healthspan such as body composition, muscle mass, strength and physical function (McLeod et al. 2016). We also hypothesized that circulating taurine levels should correlate with markers of insulin sensitivity and may also correlate with mitochondrial health. To test these hypotheses, we performed a secondary analysis of a recent study (Cefis et al. 2025) and included 49 inactive and 88 active men with ages ranging from 20 to 93. These participants were deeply phenotyped using a comprehensive battery of tests to assess physical performance (Cefis et al. 2025). Body and skeletal muscle composition were assessed using dual‐energy X‐ray absorptiometry (DXA) and peripheral quantitative computed tomography (pQCT), respectively (Cefis et al. 2025). Vastus lateralis muscle biopsies were performed to assess various aspects of mitochondrial function, including mitochondrial respiration, reactive oxygen species (ROS) production (assessed using H_2_O_2_ emission as a surrogate), and calcium handling in permeabilized myofibers (Cefis et al. 2025). Typical aging‐related changes, such as declines in skeletal muscle mass, strength, power, and physical performance, were seen in this cohort (Cefis et al. 2025). We also reported that physical activity conferred partial protection against these aging‐related declines (Cefis et al. 2025).

As shown in Figure 1A, no association between serum taurine concentration and aging was observed in our cohort. Consistent with previous studies (Graham et al. 1995; Henriksson 1991), no difference between active and inactive participants could be found (Figure 1A; active: 92.40 ± 27.04 μM, inactive: 96.93 ± 25.16 μM, *p* = 0.338). It is important to note here that serum taurine concentrations reported in Figure 1A are within the range of what was reported by Singh et al. in humans (Singh et al. 2023). No correlation was found between serum taurine concentration and the performance at the 6 min walk test, Timed Up and Go test, and 30s sit‐to‐stand test, widely used tests to assess functional capacities in humans (Marcangeli et al. 2022) (Figure 1B–D). A significant negative correlation was even observed between taurine and the performance at the step test, arguably one of the most discriminant tests to assess physical performance in older adults (Cefis et al. 2025) (Figure 1E). No association between serum taurine concentration and muscle mass, strength, or power could be observed (Figure 1F–I). No association between serum taurine concentration and body composition (total lean and fat masses) could also be observed (Figure 2A,B).

**FIGURE 1:**
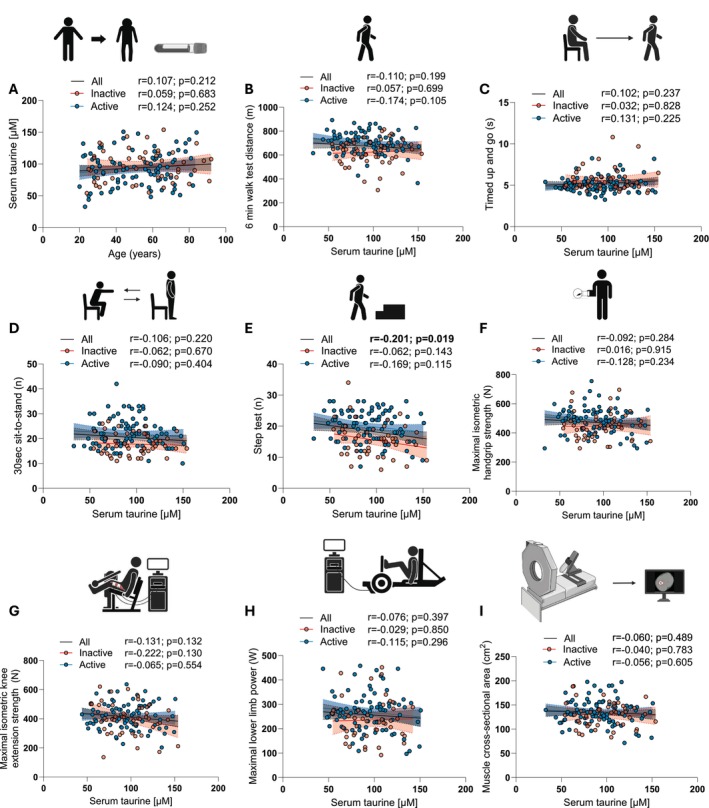
Serum taurine concentration is not associated with aging, physical performance or muscle mass and strength in humans. Correlations between circulating taurine concentration and (A) age, the performance at the (B) 6 min‐walk test, (C) Timed Up and Go test, (D) 30 s sit‐to‐stand test, (E) step test, (F) maximal isometric handgrip strength, (G) maximal isometric knee extension strength, (H) maximal lower limb power, and (I) muscle cross‐sectional area in the entire cohort (all) and in inactive and active participants. Pearson correlation coefficients (*r*) and *p*‐values are displayed above each scatter plot. *p* < 0.05 was considered statistically significant. Icons in A–H were created with biorender.com. The icon in I was created by Servier Medical Art.

**FIGURE 2:**
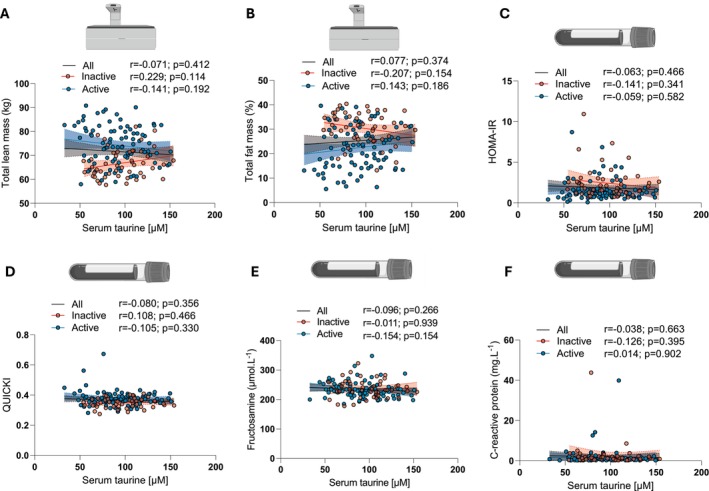
Serum taurine concentration is not associated with body composition, insulin sensitivity or the inflammatory marker CRP in humans. Correlations between circulating taurine concentration and (A) total lean mass, (B) total fat mass, (C) HOMA‐IR (D) QUICKI, (E) circulating fructosamine levels and (F) circulating C‐reactive protein (CRP) levels in the entire cohort (all) and in inactive and active participants. Pearson correlation coefficients (*r*) and *p*‐values are displayed above each scatter plot. *p* < 0.05 was considered statistically significant. Icons in A–F were created with biorender.com.

Based on findings from Singh et al. potentially linking taurine deficiency with impaired whole‐body glucose metabolism, we investigated whether serum taurine levels were associated with two widely used indices of insulin sensitivity, the Homeostatic Model Assessment of Insulin Resistance (HOMA‐IR; Matthews et al. 1985) and the Quantitative Insulin Sensitivity Check Index (QUICKI; Katz et al. 2000). As shown in Figure 2C,D, no association between serum taurine concentration and the HOMA‐IR and the QUICKI was observed. No association between serum taurine concentration and circulating fructosamine levels (a marker of glucose control) and the inflammatory marker C‐reactive protein (CRP) was also observed (Figure 2E,F, respectively).

Since Singh et al. proposed a potential link between taurine and mitochondrial health with aging, we assessed whether serum taurine concentration correlated with multiple aspects of mitochondrial function, including mitochondrial respiration, ROS production, and calcium retention capacity. As shown in Figure 3A, no correlation was observed between serum taurine concentration and maximal mitochondrial respiration rate (state III, ADP stimulated respiration). No correlation between serum taurine concentration and mitochondrial ROS production could be observed (Figure 3B,C). We previously reported that mitochondrial calcium retention capacity (mCRC) declines with aging and likely contributes to the aging‐related loss of muscle mass and strength (Cefis et al. 2025). We therefore assessed whether serum taurine concentration correlated with mCRC. As shown in Figure 3D, no correlation between serum taurine concentration and mCRC was observed.

**FIGURE 3:**
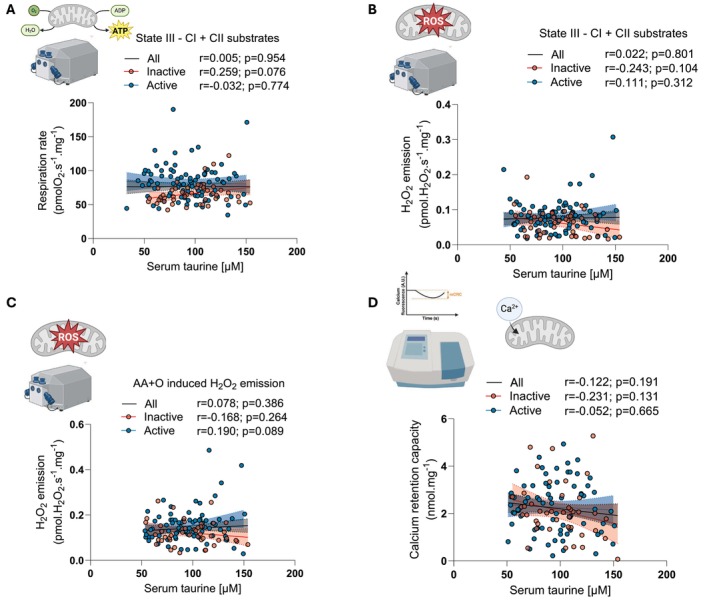
Serum taurine concentration is not associated with skeletal muscle mitochondrial respiration, ROS production or calcium retention capacity in humans. Correlations between circulating taurine concentration and mitochondrial (A) maximal (ADP‐stimulated) respiration rate driven by complex I and II substrates (glutamate, malate and succinate), (B) H_2_O_2_ emission rate (a surrogate of mitochondrial ROS production) at maximal respiration rate, (C) maximal (antimycin A‐induced) H_2_O_2_ emission rate and (D) calcium retention capacity in the entire cohort (all) and in inactive and active participants. Pearson correlation coefficients (*r*) and *p*‐values are displayed above each scatter plot. *p* < 0.05 was considered statistically significant. Icons in A–D were created with biorender.com. AA, antimycin A, O, oligomycin.

Taken altogether, our results indicate that circulating taurine levels are not associated with aging, muscle mass, strength, power, physical performance, body composition, insulin sensitivity, or mitochondrial function in humans. Our findings therefore challenge the implication of taurine deficiency as a primary driver of aging in humans, as well as its utility as a biomarker of aging and functional decline. These conclusions strengthen and extend the findings from a recent study, published while our manuscript was under review, that has challenged the implication of taurine in the aging process and its utility as an aging biomarker in mice, non‐human primates, and humans (Fernandez et al. 2025). It should, however, be noted that the present study does not rule out potential positive health impacts of taurine supplementation in older adults, especially in individuals with low circulating taurine levels and individuals with chronic diseases.

## 1 Methods

### 1.1 Participants Details and Physical Activity Assessment

Participants were recruited by advertisements in newspapers, bulletin boards, social media, and through word of mouth. To be included, participants had to be men aged between 20 and 100 years old, with a BMI between 18 and 35 kg/m^2^. The following exclusion criteria were used: uncontrolled metabolic disease, neurodegenerative disease, smoking more than 2 cigarettes per day, drinking more than 2 glasses of alcohol per day, taking anticoagulant medication, and having a pacemaker or a metal implant. Three separate on‐site visits, organized within 1 month, were required to complete all measurements and procedures. All participants provided informed written consent after having received information on the nature, goal, procedures, and risks associated with the study. Participants were informed to maintain their physical activity and dietary habits during their participation. All procedures were approved by the Ethics Committee of the *Université du Québec à Montréal* (UQÀM) (Ethic certificate number: 2020‐2703).

To estimate the status of physical activity for each participant, we combined two approaches: objective measures using a tri‐axial accelerometer (SenseWearMini Armband) 8 and self‐reported physical activity obtained through a structured interview with a trained kinesiologist. Questions asked during the interview were derived from the Physical Activity Scale for the Elderly (PASE). To be classified as active, participants had to fulfill at least one of the following criteria: (i) Engage in a minimum of 150 min per week of structured moderate to intense physical activity, (ii) Achieve a daily step count of at least 10,000 steps, or (iii) Maintain a Metabolic Equivalent of Task (MET) equal to or greater than 1.6. If participants did not meet at least one of those three criteria, they were classified as inactive.

### 1.2 Assessment of Body Composition

Total lean and fat masses were assessed using DXA (GE Prodigy Lunar). Participants were fasted for a minimum of 2 h prior to the DXA scan.

### 1.3 Assessment of Thigh Muscle Cross‐Sectional Area

Muscle thigh muscle cross‐sectional area was assessed using a peripheral quantitative computed tomography scan (p‐QCT; Stratec XCT3000 STRATEC Medizintechnik GmbH, Pforzheim, Germany, Division of Orthometrix; White Plains, NY, USA). Images were taken in the lower third of the right thigh (from the right lateral epicondyle to the greater trochanter of the right femur). Acquisition parameters were the following: voxel size: 0.5 mm; speed: 10 mm/s. p‐QCT images were analyzed using ImageJ software (NIH, Bethesda, Maryland, USA, https://imagej.nih.gov/ij/) with the plugin p‐QCT.

### 1.4 Assessment of Physical Performance

Physical performance was assessed as previously described in Marcangeli et al. (2022) using four validated tests:

- 6 min walking test (Laboratories 2002). The test was performed on a 25‐m track and participants were asked to walk as much as possible during 6 min. No encouragement was received along the test. The total distance was recorded in meters.
- Step test (Berg et al. 1992; Chung et al. 2014). Participants were placed facing toward a 20 cm height step and instructed to touch the top with the right and left foot, alternatively, as fast as possible during a 20 s period. The number of steps was recorded.
- 30 s sit‐to‐stand test. Participants were asked to repeat standing up from a sitting position and sitting down as fast as possible for 30s with arms across their chests (Yanagawa et al. 2016). The number of repetitions performed during 30s was recorded.
- Timed Up & Go test (Christopher et al. 2021). This test, which consists of standing from a chair, walking a 3 m distance, and sitting down again (Podsiadlo and Richardson 1991), was performed at a fast‐paced walking speed. The time to perform this task was recorded.

### 1.5 Assessment of Muscle Strength and Power

Maximal voluntary handgrip strength was measured using a hand dynamometer with adjustable grip (Lafayette Instrument), as previously described in (St‐Jean‐Pelletier et al. 2016). Participants stood upright with their arms at their sides, elbows extended, and palms facing their thighs. They were instructed to squeeze the hand dynamometer as hard as possible for up to 4 s. Three alternating measurements were taken for each hand, and the maximum score was recorded.

Maximal isometric knee extension strength and maximal lower limb muscle power were evaluated as previously described in Marcangeli et al. (2022). Maximal isometric knee extension strength was assessed on the right leg using a strain gauge system attached to a chair (Primus RS Chair, BTE) upon which participants were seated with the knee and hip joint angles set at 135° and 90°, respectively. The knee angle was set to 135°, compared to the typical 90°, to diminish the maximal joint torque that could be generated, particularly in light of generally more fragile bones in older adults. The tested leg was fixed to the lever arm at the level of the lateral malleoli on an analog strain gauge to measure strength. The highest of three maximum voluntary contractions was recorded.

Lower limb muscle power was measured on the right leg using the Nottingham Leg Extensor Power rig in a sitting position (Bassey and Short 1990). Participants were asked to push the pedal down as hard and fast as possible, accelerating a flywheel attached to an analog‐toto‐digital converter. Power was recorded for each push until a plateau/decrease was observed.

### 1.6 Blood Preparation and General Blood Biochemistry

Fifteen ml of venous blood samples were collected after an overnight fast in BD Vacutainer SST Tube with Hemogard Closure (Gold, Dufort et Lavigne Ltée, Qc, Canada, BEC367986). Tubes were inverted 6 times and were then left at room temperature for a minimum of 30 min and a maximum of 60 min for clotting. Gold tubes were then stored at 4°C for a maximum of 4 h before centrifugation. Gold tubes were centrifuged at 2000 *g* for 15 min at room temperature. After centrifugation, the supernatant (serum) was collected and aliquoted into 1.5 mL Eppendorf tubes and immediately stored at −80°C until use.

Serum glucose concentrations were assessed using a coupled enzymatic assay in a Beckman Coulter AU analyzer according to the manufacturer's instructions (Beckman Coulter, BAOSR6X21). Serum insulin concentrations were assessed using the ARCHITECT Insulin assay according to the manufacturer's instructions (Abbott, ARCHITECT Insulin 8K41). Fructosamine levels were assessed using a colorimetric assay according to the manufacturer's instructions (Randox, FR 3133). Total cholesterol and HDL‐cholesterol were assessed using a Beckman Coulter AU analyzer according to the manufacturer's instructions (Beckman Coulter, BAOSR6X16 and BAOSR6x95, respectively). LDL cholesterol was calculated based on HDL‐cholesterol and triglyceride values as follows: LDL cholesterol = total cholesterol—HDL−(triglycerides/5). The HOMA‐IR was calculated as [fasting glucose (mmol/l) × fasting insulin (pmol/L)]/135 (Matthews et al. 1985). The Quantitative Insulin Sensitivity Check Index (QUICKI) 1 / [log fasting insulin (μU/mL) + log glucose (mg/dL)] (Katz et al. 2000). C‐reactive protein (CRP) levels were assessed using the Beckman Coulter CRP latex assay in a Beckman Coulter AU analyzer according to the manufacturer's instructions (Beckman Coulter, BAOSR6X99). Abnormally high CRP values for two participants were removed from the analyses.

### 1.7 Taurine Assessment

Fasting serum taurine levels were measured at the metabolomics platform of the Centre de Recherche du Centre Hospitalier de l'Université de Montréal (CR‐CHUM) using an isotope dilution LC‐ESI‐MS/MS method. Briefly, each sample (20 μL) was deproteinized using an acidified acetonitrile solution (final 81% acetonitrile, 18 mM ammonium formate, pH 3.0) containing 10 μM taurine‐d4 (CDN Isotopes, Pointe‐Claire, Canada) as an internal standard. Samples were incubated at 4°C for 15 min with agitation and then centrifuged (4°C, 10 min, 20,000 g). This procedure was also applied to calibrators and a blank. The supernatant (2 μL) was analyzed on a Shimadzu Nexera X2 HPLC system equipped with a Poroshell column (120 HILIC‐Z, 2.1 × 100 mm, 2.7 μm) and a guard column (120 HILIC‐Z, 2.1 × 5 mm, 2.7 μm) thermostatically controlled at 30°C. The following gradient was used at a flow rate of 0.8 mL/min: 0 min = 100% B, 10 min = 70% B, 11 min = 100% B up to 16 min: A = 20 mM ammonium formate in water, pH 3.0; B = 20 mM ammonium formate in 9:1 acetonitrile: water, pH 3.0. A QTrap 6500 system (SCIEX) was used for the detection of positive ions using the “scheduled MRM” mode. The transitions used were 126.0/107.9 for taurine and 129.9/112.0 for taurine‐d4. The ratio of the peak area for taurine (external standard) to the peak area for taurine‐d4 (internal standard) was used for quantification. Samples were analyzed randomly in five different series. Each one included a random selection of young and old participants. In addition, the serum of a young and old participant was analyzed at the beginning and the end of each series. The intra‐assay coefficient of variation (CV) of serum taurine obtained from a young and old participant was 0.0%–2.2% and 0.6%–2.8%, respectively. The intra‐assay CV of taurine sample duplicates was 0.0%–5.2%. The inter‐assay CV was assessed using the ratios of external/internal taurine standards obtained for low, mid, and high concentrations of taurine standards and was 18%, 7%, and 14%, respectively.

### 1.8 Skeletal Muscle Biopsies

Muscle biopsies were collected in the vastus lateralis using a suction‐modified Bergström needle under local anesthesia (lidocaine injection) as extensively detailed in (Cefis et al. 2025). Part of the tissue collected during this procedure was immediately prepared to assess mitochondrial function.

### 1.9 Preparation of Permeabilized Muscle Fibers for In Situ Assessment of Mitochondrial Function

Once dissected, muscle biopsy samples were weighed with a precision scale and then rapidly immersed in ice‐cold stabilizing buffer A (2.77 mM CaK_2_ ethylene glycol‐bis‐(2‐aminoethylether)‐N,N,N,N‐tetraacetic acid (EGTA), 7.23 mM K_2_ EGTA, 6.56 mM MgCl_2_, 0.5 mM dithiothreitol (DTT), 50 mM 2‐(N‐morpholino) ethanesulfonic acid potassium salt (K‐MES), 20 mM imidazole, 20 mM taurine, 5.3 mM Na_2_ ATP, and 15 mM phosphocreatine, pH 7.3). Muscle biopsy samples were separated into small fiber bundles using fine forceps under a surgical dissecting microscope (Leica S4 E, Germany). Muscle fiber bundles were incubated in a glass scintillation vial for 30 min at low rocking speed containing buffer A supplemented with 0.05 mg/mL saponin (Sigma‐Aldrich) to selectively permeabilize the sarcolemma. Fiber bundles were divided into two parts; one was washed 3 times for 10 min at low rocking speed in the MiR05 buffer (110 mM sucrose, 20 mM HEPES, 10 mM KH_2_PO_4_, 20 mM taurine, 60 mM K‐lactobionate, 3 mM MgCl_2_, 0.5 mM EGTA, 1 g/L of fatty acid free BSA, pH 7.4) to assess mitochondrial respiration and H_2_O_2_ emission. The other part of the fiber bundles was washed 3 times for 10 min at low rocking speed in the C buffer (80 mM K‐MES, 50 mM HEPES, 20 mM taurine, 0.5 mM DTT, 10 mM MgCl_2_, 10 mM ATP, pH 7.3) to assess mitochondrial calcium retention capacity. All incubations were performed in vials placed on ice.

### 1.10 Assessment of Mitochondrial Respiration

Permeabilized myofibers from muscle biopsy samples were used to assess mitochondrial respiration in an Oroboros O2K high‐resolution fluororespirometer (Oroboros Instruments, Austria) at 37°C in 2 mL of MiR05 buffer. Briefly, 3–6 mg (wet mass) of permeabilized fiber bundles were weighed and added to the respiration chambers. Mitochondrial respiration was assessed using the following sequential addition of substrates and inhibitors: 10 mM glutamate + 5 mM malate, 2 mM ADP, 10 mM succinate, 1 μM oligomycin, and 2 μM antimycin A. Respiration rates were normalized as picomoles of dioxygen per second per mg of wet muscle mass. All respiration experiments were analyzed with MitoFun (https://zenodo.org/records/7510439), a homemade code to analyze mitochondrial function data in the Igor Pro 8 software (Wavemetrics, OR, USA).

### 1.11 Assessment of Mitochondrial H_2_O_2_ Emission

The H_2_O_2_ emission from myofiber bundles was assessed by monitoring the rate of H_2_O_2_ release using the Amplex Ultra Red‐horseradish peroxidase system. This was performed along with respiration assessment in the Oroboros O2K high‐resolution fluororespirometer (Oroboros Instruments, Austria) at 37°C in 2 mL of MiR05 buffer supplemented with Amplex Ultra Red (10 μM), SOD (5 U/mL), and HRP (1 U/mL). A calibration curve was generated daily using successive additions of known [H_2_O_2_] in the absence of tissue. H_2_O_2_ emission was normalized as picomoles per second per milligram of wet muscle mass. All H_2_O_2_ emission experiments were analyzed with MitoFun (https://zenodo.org/records/7510439), a homemade code to analyze mitochondrial function data in the Igor Pro 8 software (Wavemetrics, OR, USA).

### 1.12 Assessment of Mitochondrial Calcium Retention Capacity

The mitochondrial calcium retention capacity was then determined in permeabilized phantom fibers as previously described (Cefis et al. 2025; Gouspillou et al. 2014; Picard et al. 2008). Briefly, a muscle bundle of 3–6 mg wet mass was added to 600 μL of CRC buffer containing ~30 μM of Ca^2+^ supplemented with 5 mM glutamate, 2.5 mM malate, 10 mM inorganic phosphate (P_i_), 1 μM calcium‐green 5 N, and 0.5 μM oligomycin. Mitochondrial Ca^2+^ uptake was immediately followed by monitoring the decrease in extramitochondrial Ca^2+^concentration using the fluorescent probe calcium‐green 5 N (C3737, ThermoFisher). Fluorescence was detected using a spectrophotometer (Hitachi F2500, FL Solutions software) with excitation and emission detectors set at 505 and 535 nm, respectively. All measurements were performed at 37°C. Progressive uptake of Ca^2+^ by mitochondria was monitored until mitochondrial Ca^2+^ release caused by the opening of the mitochondrial permeability transition pore (mPTP) was observed as the inversion of the signal. Mitochondrial calcium retention capacity was calculated as the total amount of Ca^2+^ taken by mitochondria before Ca^2+^ release. Fluorescent units were converted into Ca^2+^ concentration using a standard curve obtained daily using successive additions of known Ca^2+^ concentration in the absence of a sample. CRC values were expressed per milligram of wet fiber mass. All mitochondrial calcium retention capacity experiments were analyzed with MitoFun (https://zenodo.org/records/7510439), a homemade code to analyze mitochondrial function data in the Igor Pro 8 software (Wavemetrics, OR, USA).

### 1.13 Statistical Analyses

All statistical analyses were performed using GraphPad Prism 10.1.3. For all correlation analyses, simple linear regressions were performed, and Pearson *r* coefficients were calculated. Individual data are displayed in each graph. *p* < 0.05 was considered significant.

## Author Contributions

V.M., M.C., and G.G. designed this secondary analysis. P.G., M.A.‐L., M.B., R.R., J.A.M., and G.G. designed the overall research project and secured funding. M.C., V.M., R.H., J.G., J.P.L.G., P.G., J.A.M., and G.G. performed the experiments. All authors contributed to data analysis and interpretation. V.M., M.C., and G.G. wrote the original manuscript. All authors have read and approved the manuscript.

## Conflicts of Interest

The authors declare no conflicts of interest.

## Data Availability

The data that support the findings of this study are available on request from the corresponding author. The data are not publicly available due to privacy or ethical restrictions.
